# Ergonomic Implants: A Single Centre Experience in Post-Mastectomy Breast Reconstruction

**DOI:** 10.3390/medicina62061064

**Published:** 2026-05-31

**Authors:** Daniele Fusario, Sofia Alessandrini, Gianfranco Lolli

**Affiliations:** Breast Surgery Unit, Department of Surgery, San Giovanni Battista Hospital, 06034 Foligno, Italy; sofia.alessandrini@uslumbria2.it (S.A.); gianfranco.lolli@uslumbria2.it (G.L.)

**Keywords:** implant-based breast reconstruction, ergonomic implants, mastectomy, breast surgery

## Abstract

*Background and Objectives*: Implant-based breast reconstruction is nowadays the most commonly used reconstructive technique and offers a safe and relatively simple surgical approach. Textured, anatomically shaped implants have been the primary option for women undergoing breast reconstruction for decades. However, anatomically shaped implants are form-stable devices, providing firmness and rigidity, and can cause malrotation, requiring revision surgery. Ergonomic round silicone gel implants began to gain popularity due to the capacity to adapt to gravity and the patient’s position, providing a natural, “anatomical” appearance in an upright position. The objective of this study was to describe our two-year experience with Motiva Ergonomix Round Silksurface™ implants and to evaluate their safety and efficacy for immediate and delayed breast reconstruction procedures. *Materials and Methods*: An observational retrospective study was conducted on a population of patients undergoing mastectomy for breast cancer and immediate or delayed reconstruction with Motiva Ergonomix Round Silksurface™ implants. We enrolled 61 patients from December 2023 to December 2025: a total of 87 ergonomic prostheses were implanted. *Results*: The average implant volume was 269 ± 105 (105–510) cc. One surgical infection occurred (1.1%); three persistent seromas (3.4%); and two cases of rippling (2.3%). Only one CTCAE G3 complication was recorded, a grade IV Baker capsular contraction (1.1%). The BREAST-Q results after surgery showed a high rate of patient satisfaction. *Conclusions*: Ergonomic round implants with a nano-textured surface were developed to give a natural breast appearance following mastectomy, as well as to prevent rotation, one of the complications that can affect anatomical implants. Our data have demonstrated low complication rates and high patient satisfaction. These results encourage us to expand our experience with these ergonomic, round, nano-textured implants and seriously consider them as an increasingly important device in breast reconstruction after mastectomy to be placed alongside more well-known anatomical implants.

## 1. Introduction

Breast cancer is the most common female cancer in the European Union [1], with variable incidence across countries but an overall lifetime risk of 1 in 10 for women [2]. Breast cancer mortality rates declined in the European Union and are predicted to fall further mainly due to improvements in the management and treatment of cancer, as well as early diagnosis and the finest screening programme [3].

Increased survival results in an increasing importance of ‘quality of life’ measures as benchmarks of therapeutic success, as opposed to mortality rates alone.

Nowadays, implant-based breast reconstruction is the most commonly used reconstructive technique and offers a safe and relatively simple approach [4]. In a recent multicentric study on the state of breast reconstruction, the prosthetic reconstruction was 92.2% of all procedures, and of these, Direct to Implant (DTI) was 27.6% and two-stage breast reconstruction was 64.6% [5]. Traditionally, during mastectomy, a pocket is developed either between the subcutaneous tissue and the pectoralis major muscle or total submuscular/partial submuscular [6,7,8,9].

The first silicone gel-filled breast implant was introduced in 1962 by Dr. Thomas Cronin and Dow Corning [10]. The first use of silicone breast implants in reconstructive surgery was with the work of Snyderman and Guthrie in 1971 [11], who positioned the implants under the pectoralis major muscle, preserving the anatomical integrity of the pectoralis major. Due to the high complication rate, including implant exposure, capsular contracture, and implant dislocation [12], this technique fell into disuse.

Subsequent decades have seen significant improvements in implant design, such as the elimination of fixation points, the development of cohesive gels, and the reduction in gel loss, increasing their durability and safety.

Breast reconstruction with implants has undergone a dramatic improvement in the last decade, thanks to the reintroduction of prepectoral implant placement, as well as following the introduction of Acellular Dermal Matrices (ADMs) and synthetic mesh, broadening the range of reconstructive strategies and expanding the indications for immediate reconstruction. Continued advances in both oncological and reconstructive surgery, particularly in skin- and nipple-sparing mastectomy techniques, have led to a renewed interest in the prepectoral plane after decades of predominantly subpectoral breast reconstruction.

In the 1990s, due to the Food and Drug Administration (FDA) moratorium on silicone implants and their potential autoimmune risks, saline implants gained popularity. The potential autoimmune risks were refuted in 2000 by the Institute of Medicine’s review, which found no causal link between implants and systemic diseases [10].

Ongoing research into materials has led to the development of more cohesive silicone gels and different surface textures and shapes.

Textured, anatomically shaped implants have been the primary option for women undergoing breast reconstruction for decades due to their ability to maintain the shape and appearance of a natural breast [13]. However, anatomically shaped implants are form-stable devices that provide firmness and rigidity and can cause malrotation, requiring revision surgery. Furthermore, the discovery of a possible link between textured shell implants and BIA-ALCL [14] led to the withdrawal of Allergan’s cohesive, stable, textured implants.

Therefore, breast implants with minimal textures began to gain popularity, although implants with a smooth surface were reported to have increased rates of capsular contracture (reported as 15.56% in some cases, compared to 3.8–5.27% for textured implants [15]), the migration of the device due to a lack of tissue adhesion, and the impossibility of having an anatomically shaped implant.

Recently, the definition of breast implant illness has animated scientific debate on modern implants, although the available evidence does not demonstrate a significant association between implants and systemic symptoms [10].

Establishment Labs (Alajuela, Costa Rica) launched the Motiva Ergonomix implants in 2013. These round silicone gel implants adapt to gravity and the patient’s position, providing a natural “anatomical” appearance in an upright position [16,17,18,19]. This is due to the combination of a specific elastomeric shell and the rheological properties of the silicone gel [17,19]. The nanotexturization of the surface, according to the surface characteristics scale, classifies them as the lowest grade [20], thus with a very low risk of developing BIA-ALCL. Currently, no cases of BIA-ALCL have been reported with Motiva implants [21]. Reported rates of capsular contracture with Motiva implants in breast augmentation are less than 2% [21,22].

The Motiva Ergonomix implants present four possible projections of MINI, DEMI, FULL, and CORSE, from the least to the most projecting.

To date, to the best of our knowledge, only two studies have been published on the use of these implants in breast reconstruction following mastectomy [23,24].

The objective of this retrospective study was to describe our two-year experience with Motiva Ergonomix implants and to evaluate their safety and efficacy for immediate and delayed breast reconstruction procedures, as well as patient satisfaction.

## 2. Materials and Methods

We designed an observational retrospective study conducted on a population of patients undergoing direct or delayed reconstruction with Motiva Ergonomix Round Silksurface™ implants following mastectomy for cancer or BRCA mutation in the Breast Surgery Unit, Azienda USL Umbria 2 (Foligno, Italy) from December 2023 to December 2025. Before surgery, all the patients were informed about the difference between ergonomic implants and anatomical implants, and a sizer of ergonomic implant was shown: the patients enrolled accepted the possible use of ergonomic implants (an intraoperative and definitive evaluation based on flap thickness and perfusion, lymph node status, and global symmetry was performed by the first surgeon).

Inclusion criteria were breast cancer or BRCA1/2-mutated patients who underwent immediate or delayed implant-based reconstruction after mastectomy. Exclusion criteria were reconstructions with different implants. Details of patient’s enrolment are reported in Supplementary File S1.

All the procedures performed in this study were in accordance with the ethical standards of the national research committee and with the 1964 Helsinki declaration and its later amendments. The devices were used according to the label’s indication and did not require any modification of the standard therapeutic protocols. Informed consent was obtained from all patients included in the study before surgery.

Enrolment started in December 2023 and ended in December 2025. The same surgeons (GL, SA, DF) performed all the procedures.

Nipple- or skin-sparing mastectomy (NSM/SSM) was performed through an inframammary, lateral, or mammary fold incision, and skin flaps were created in the subcutaneous layer. Once the breast gland was removed, the retro-areolar tissue was removed, preserving the skin of the nipple-areola complex (NAC) if the extemporaneous histological examination was negative. Based on the thickness of the mastectomy skin flap, assessed by intraoperative measurement and on the factors influencing flap vascularity (smoking status, BMI, diabetes, prior radiotherapy, prior surgery), the appropriate prosthetic plane was chosen between prepectoral, subpectoral, or dual plane and the type of reconstruction: immediate or two-stage. For subpectoral reconstruction, the prosthetic pocket was prepared, selective denervation of the pectoralis major muscle was performed, the prosthesis was placed, and the pocket was closed with absorbable sutures. For prepectoral or dual-plane reconstruction, the prosthesis was covered with a bovine pericardial ADM (Tutomesh^®^, Tutogen Medical, Neunkirchen am Brand, Germany) and rehydrated for 15 min in saline. The medial and lateral edges of the ADM were fixed to the muscle fascia with absorbable sutures. Before implantation, the prosthetic pockets were washed with povidone iodine. The prosthetic implants were immersed in a solution of saline and gentamicin. A drain was placed in the prosthetic pocket, and patients received intravenous antibiotics for the first 24 h and oral antibiotics until the surgical drains were removed.

The outcomes of this study were to evaluate complication and reoperation rates, aesthetic outcome, patient satisfaction, and quality of life.

We recorded clinical and operative characteristics: age, BMI, primary or secondary operation, implant size, and operative plane. Postoperative complications were divided into minor and major complications (requiring intervention), according to the Common Terminology Criteria for Adverse Events (CTCAE), version 4.0 [25].

In January 2026, a subjective evaluation was conducted using the postoperative section of BREAST-Q (Memorial Sloan-Kettering Center and The University of British Columbia © 2006). As reported in a recent study on long-term patient-reported QOL after breast implant reconstruction [26], the BREAST-Q reconstruction survey was divided into multiple independent scales: satisfaction with breasts (16 items), satisfaction with the outcome (7 items), psychosocial well-being (10 items), physical well-being (16 items), and sexual well-being (6 items). For each scale, item responses were summed up and transformed into a score, ranging from 0 to 100. Higher scores indicated greater satisfaction or QOL.

The BREAST-Q scores for each patient were converted from survey scores (1 through 5) to a continuous range from 0 to 100 the Q Score Scoring Software (Ver 2.0), with a higher score indicating greater satisfaction or better HR-QOL.

Categorical variables were reported as numbers and percentages. Continuous variables were reported as mean and standard deviation (SD) or median and interquartile range (IQR), depending on the normal distribution of data. We verified the normal distribution of continuous variables using the Shapiro–Wilk test. Where applicable, group comparisons were conducted for continuous variables using Student’s *t*-test and for discrete variables using the χ^2^ test. A two-tailed *p*-value of <0.05 was considered statistically significant. SPSS software, Ver. 27.0 (IBM Corp., Armonk, NY, USA) was used for statistics.

## 3. Results

We enrolled 61 patients who underwent mastectomy in our breast unit from December 2023 to December 2025: a total of 87 ergonomic prostheses were implanted.

The characteristics of the study’s population are summarised in Table 1: median age was 51 yo, average BMI was 22.5, and average follow-up time was 17 (range 1–29) months.

A mastectomy was performed for Breast Cancer Gene mutation (BRCA) presence in five patients (8.2%), ductal carcinoma in situ in 14 patients (23%), and invasive ductal carcinoma in 42 patients (68.8%).

Indications for second-time reconstruction were tissue expander substitution in 13 patients, implant rupture in eight patients, and anatomical implant rotation in six patients (some patients had more than one indication for surgery).

Mastectomies were performed by S Italic incision in 11 breasts (12.6%), by peri-areolar incision in 37 breasts (42.5%) and by sub-mammary fold incision in 39 breasts (44.9%), as reported in Table 2.

**Figure 1:**
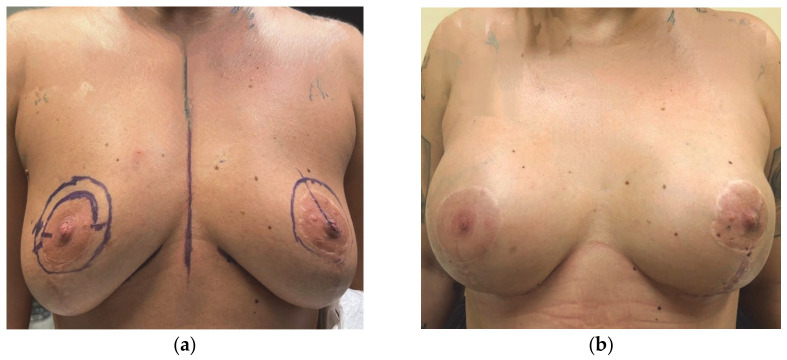
Bilateral NSM for BRCA 2 mutation with immediate prepectoral breast reconstruction with Motiva Ergonomix Round Silksurface, FULL profile, in a patient previously treated with bilateral oncoplastic procedure. (**a**) Preoperative breasts; (**b**) one month follow-up.

Bilateral reconstruction was performed in 26 patients and a unilateral reconstruction in 35; an immediate symmetrisation was performed in 18 patients who underwent unilateral mastectomy (Figure 1).

Implants were placed in the prepectoral plane in 11 breasts (12.6%) and covered by ADM Tutomesh^®^ (Tutogen Medical, Germany); in the subpectoral plane in 67 breasts (77%); and a dual-plane placement reinforced by ADM Tutomesh^®^ (Tutogen Medical, Germany) was used in nine breasts (10.4%).

The average implant volume was 269 ± 105 (105–510) cc; the projection was MINI in 33 implants, DEMI in 45 implants, FULL in eight implants, and CORSE in one implant.

At 30 days after surgery, one surgical infection occurred (1.1%) that was treated with antibiotic therapy; three persistent seromas (3.4%), meaning a long-lasting serous fluid drainage or collection needing aspiration from the mastectomy site [27]; and two cases of rippling (2.3%). In this series, no cases of flipping or malrotation were recorded. In only one case, we recorded a CTCAE G3 complication, a grade IV Baker capsular contraction due to an abnormal retraction of an ADM that was not well-integrated following radiotherapy.

Complications were stratified by group based on the type of reconstruction used (prepectoral, dual pane, or subpectoral). Within the groups, further stratification was performed, distinguishing between DTI reconstructions and second-stage reconstructions. No statistically significant differences emerged from the analyses conducted. Detailed results are reported in Table 3.

### Measure of HRQOL and Aesthetic Outcome

All the patients answered the five domains of the survey. Table 4 shows these results. The survey was administered during a follow-up visit 1 month after surgery. The patients reported a high level of satisfaction with the outcomes in each subgroup; no statistically significant difference was found.

## 4. Discussion

Breast reconstruction at the same time of mastectomy for breast cancer is nowadays a recommended practice with optimal aesthetic results for patients and surgeons. After the introduction of synthetic devices and ADMs, which can create an additional layer between the prosthesis and subcutaneous tissue, the prepectoral approach has regained a central role as an alternative to the submuscular approach in breast reconstruction.

Consequently, implants and their characteristics have assumed ever greater importance.

Furthermore, the relatively recent discovery of a possible link between textured shell implants and BIA-ALCL has increasingly pushed the use of nano-textured or smooth implants.

Today, breast implant technology is highly advanced, with CE- and FDA-approved manufacturers offering implants with high safety, durability, and warranties. However, today’s implants are never permanent, frequently requiring revision or replacement over time. To provide high-quality, evidence-based, patient-centred care, a deeper understanding of breast implant materials, surgical techniques, and safety profiles is essential.

This study aimed to analyse the two-year experience of using ergonomic prostheses in breast reconstruction following mastectomy, highlighting the outcomes and the patient’s satisfaction rate.

Motiva Ergonomix Round Silksurface™ is a round implant with a specific elastomeric shell featuring the unique rheological properties of a newly developed silicone gel that adapts to the patient’s position after implantation, following gravity, for a more natural look and feel, without the risk of rotation that plagues anatomical implants [28]. The surface of Motiva implants, called Silksurface, features nanotexturing that allows for a more natural interaction between the implant and the surrounding tissue, allowing the implant to adapt to the breast’s movements.

In this cohort of patients, the early BREAST-Q results after surgery showed a high rate of patient satisfaction with the reconstruction, the implant, and high physical well-being scores; these results were in line with, if not superior to, other case studies reported in the literature [29].

Analysing the complications that occurred in this cohort of patients, it appears evident that the total complications (7.9%) are in line with those reported in the literature for this type of prosthesis [22,23,24] and lower than those in other cases that used common anatomical prostheses [30,31,32,33,34]. Specifically, the most evident data is the significant reduction in CTCAE grade 3–4 complications requiring re-operation, which in our case series concern only one patient (1.1%) with prepectoral reconstruction and implant covered with bovine pericardial ADM who underwent radiotherapy, following which an abnormal contracture and retraction of the dermal matrix developed; in this case, the reconstruction did not fail, as we proceeded with the removal of the ADM and prosthetic replacement.

Among minor complications, we recorded a low rate of infections (1.1%) compared to other experiences in the use of these prostheses [23,24], which were attributable to the scrupulous aseptic procedure adopted in the operating room and the use of advanced dressings in the case of patients at high risk of infection [35].

Despite the use of drains and postoperative compressive dressings to narrow the prosthetic pocket, we recorded 3.4% of persistent seromas, which is in line with other published data [22].

We had noticed only 2.3% of rippling with palpable or visible folds on the surface of the reconstructed breast in the upper quadrants, due to a very thin skin mastectomy flap with no pain or capsule contracture associated. This data are lower than that reported in other experiences in the literature [36,37].

No case of malrotation was recorded as ergonomic round prostheses, as their design and shape inherently cannot cause malrotation; however, this is problematic due to the “teardrop” shape with which anatomical prostheses are designed.

Analysing the literature, only two other case studies have been published on the use of this type of ergonomic prosthesis in reconstruction following mastectomy.

Limitations of this study include the short follow-up period and the early results of BREAST-Q, the small population studied, and its retrospective nature.

Nevertheless, the data collected, the patient satisfaction rate, and the clinical judgement increasingly support the use of these ergonomic prostheses, so that we may be able to produce more robust evidence in the near future.

## 5. Conclusions

The quest for an ever-improving and more natural breast appearance following mastectomy has led to ongoing research into “natural” materials and implants over the years, which is always subject to clinical monitoring and patient satisfaction assessments.

Knowledge of the evolution and innovations related to prosthetic implants is essential for both the scientific community and policymakers, as it can influence the effectiveness of future studies and provide insights into the role of implants in reconstructive surgery, while also highlighting their potential critical issues.

Ergonomic round implants with a nano-textured surface were developed precisely for this purpose, as well as to prevent rotation, one of the complications that can affect anatomical implants.

To the best of our knowledge, this is one of the very first reported experiences with the use of these implants in breast reconstruction, and our data have demonstrated low complication rates and high patient satisfaction. These results encourage us to expand our experience with these ergonomic, round, nano-textured implants and seriously consider them as an increasingly important device in breast reconstruction after mastectomy to be placed alongside to the more well-known anatomical implants.

## Figures and Tables

**Table 1:** Characteristics of the study’s population.

Number of Patients		61
Implants	Mono-lateral	35
	Bilateral	26
	Total	87
Age	(median)	51 (22–75)
BMI	(median)	22.5 ± 2.6
Reconstruction	Immediate	67 (77%)
	Second Reconstructive stage	20 (23%)
Symmetrisation		44 (72%)

**Table 2:** Surgical data.

Implant volume	(cc)	245 (10–510)
Skin Incision	Peri-areolar	37 (42.5%)
	Sub-mammary fold	39 (44.9%)
	S Italic	11 (12.6%)
Projection	MINI	33 (38%)
	DEMI	45 (51.7%)
	FULL	8 (9.2%)
	CORSE’	1 (1.1%)
Implant Placement	Prepectoral	11(12.6%)
	Dual plane	9 (10.4%)
	Subpectoral	67 (77%)

**Table 3:** Surgical and aesthetic complications.

Entire Series (87)		Prepectoral (11)		Dual Plane (9)		Subpectoral (67)		*p*
		DTI 10 (91%)	ST 1 (9%)	DTI 9 (100%)	ST 0 (0%)	DTI 48 (72%)	ST 19 (28%)	
Complications (total)	7 (7.9%)	1 (10%)	-	1 (11.1%)	-	4 (8.4%)	2 (10.4%)	0.233
SSI (surgical site infection)	1 (1.1%)	-	-	-	-	1 (2.1%)	-	0.734
Seroma	3 (3.4%)	-	-	-	-	2 (4.2%)	1 (5.2%)	0.839
Capsular Contraction	1 (1.1%)	1 (10%)	-	-	-	-	-	0.354
Rippling	2 (2.3%)	-	-	1 (11.1%)	-	1 (2.1%)	1 (5.2%)	0.116
G3-4 CTCAE Complications	1 (1.1%)	1 (10%)	-	-	-	-	-	0.284

DTI: direct to implant; ST: second-time reconstruction.

**Table 4:** BREAST-Q results.

Entire Series (87)		Prepectoral (11)		Dual Plane (9)		Subpectoral (67)		*p*
		DTI 10 (91%)	ST 1 (9%)	DTI 9 (100%)	ST 0 (0%)	DTI 48 (72%)	ST 19 (28%)	
Satisfaction withbreasts	76.8 ± 12.1	77 ± 12.1	73.8 ± 9.3	72.4 ± 9.9	-	78 ± 11.9	73 ± 10	0.36
Psychosocial wellness	77.2 ± 12.3	76.1 ± 11.7	77.2 ± 12.3	77.6 ± 12	-	79.2 ± 14.2	76.6 ± 12	0.75
Sexual wellness	64.3 ± 13.9	64.3 ± 13.9	60.7 ± 13.1	65.1 ± 11.2	-	65.3 ± 14.7	64.8 ± 12.9	0.53
Physical impact	58.8 ± 12.0	57.5 ± 14.4	58.8 ± 12	75.3 ± 12.4	-	58.8 ± 11.8	56.5 ± 13.2	0.43
Satisfaction with outcome	74.7 ± 10.6	74.7 ± 10.6	72.5 ± 11.8	74.2 ± 11.7	-	75 ± 10.7	73 ± 12.1	0.15

DTI: direct to implant; ST: second-time reconstruction.

## Data Availability

The original contributions presented in this study are included in the article/Supplementary Material. Further inquiries can be directed to the corresponding author.

## Supplementary Materials

The following supporting information can be downloaded at: https://www.mdpi.com/article/10.3390/medicina62061064/s1, Supplementary File S1: Details of patient’s enrolment.

## Abbreviations

The following abbreviations are used in this manuscript:

CTCAE	Common Terminology Criteria for Adverse Events
DTI	Direct to Implant
ADM	Acellular Dermal Matrix
QOL	Quality of Life
BIA-ALCL	Breast Implant-Associated Anaplastic Large Cell Lymphoma

## References

[1] Biganzoli L., Cardoso F., Beishon M., Cameron D., Cataliotti L., Coles C.E., Bolton R.C.D., Trill M.D., Erdem S., Fjell M. (2020). The requirements of a specialist breast centre. Breast.

[2] Publications Office of the European Union (2006). European Guidelines for Quality Assurance in Breast Cancer Screening and Diagnosis.

[3] Carioli G., Malvezzi M., Rodriguez T., Bertuccio P., Negri E., La Vecchia C. (2017). Trends and predictions to 2020 in breast cancer mortality in Europe. Breast.

[4] Albornoz C.R., Bach P.B., Mehrara B.J., Disa J.J., Pusic A.L., McCarthy C.M., Cordeiro P.G., Matros E.M. (2013). A paradigm shift in U.S. Breast reconstruction: Increasing implant rates. Plast. Reconstr. Surg..

[5] Casella D., Calabrese C., Orzalesi L., Gaggelli I., Cecconi L., Santi C., Murgo R., Rinaldi S., Regolo L., Amanti C. (2017). Current trends and outcomes of breast reconstruction following nipple-sparing mastectomy: Results from a national multicentric registry with 1006 cases over a 6-year period. Breast Cancer.

[6] Nahabedian M.Y., Cocilovo C. (2017). Two-Stage Prosthetic Breast Reconstruction: A Comparison Between Prepectoral and Partial Subpectoral Techniques. Plast. Reconstr. Surg..

[7] Casella D., Calabrese C., Bianchi S., Meattini I., Bernini M. (2015). Subcutaneous tissue expander placement with synthetic titanium-coated mesh in breast reconstruction: Long-term results. Plast. Reconstr. Surg. Glob. Open.

[8] Casella D., Di Taranto G., Marcasciano M., Lo Torto F., Barellini L., Sordi S., Gaggelli I., Roncella M., Calabrese C., Ribuffo D. (2019). Subcutaneous expanders and synthetic mesh for breast reconstruction: Long-term and patient-reported BREAST-Q outcomes of a single-center prospective study. J. Plast. Reconstr. Aesthet. Surg..

[9] Marcasciano M., Frattaroli J., Mori F.L.R., Lo Torto F., Fioramonti P., Cavalieri E., Kaciulyte J., Greco M., Casella D., Ribuffo D. (2019). The New Trend of Pre-pectoral Breast Reconstruction: An Objective Evaluation of the Quality of Online Information for Patients Undergoing Breast Reconstruction. Aesthet. Plast. Surg..

[10] Patel B., Szymanski K., Schaffner A. (2026). Breast Implants.

[11] Snyderman R.K., Guthrie R.H. (1971). Reconstruction of the female breast following radical mastectomy. Plast. Reconstr. Surg..

[12] Nahai F., Bostwick J. (1982). Aesthetic aspects of breast reconstruction. Aesthet. Plast. Surg..

[13] Calobrace M.B., Capizzi P.J. (2014). The biology and evolution of cohesive gel and shaped implants. Plast. Reconstr. Surg..

[14] Santanelli di Pompeo F., Sorotos M. (2018). EURAPS Editorial: BIA-ALCL, a brief overview. J. Plast. Reconstr. Aesthet. Surg..

[15] Shauly O., Gould D.J., Patel K.M. (2019). Microtexture and the Cell/Biomaterial Interface: A Systematic Review and Meta-Analysis of Capsular Contracture and Prosthetic Breast Implants. Aesthet. Surg. J..

[16] Mendonça Munhoz A., Santanelli di Pompeo F., De Mezerville R. (2017). Nanotechnology, nanosurfaces and silicone gel breast implants: Current aspects. Case Rep. Plast. Surg. Hand Surg..

[17] Sforza M., Zaccheddu R., Alleruzzo A., Seno A., Mileto D., Paganelli A., Sulaiman H., Payne M., Maurovich-Horvat L. (2018). Preliminary 3-Year Evaluation of Experience With SilkSurface and VelvetSurface Motiva Silicone Breast Implants: A Single-Center Experience With 5813 Consecutive Breast Augmentation Cases. Aesthet. Surg. J..

[18] Doloff J.C., Veiseh O., de Mezerville R., Sforza M., Perry T.A., Haupt J., Jamiel M., Chambers C., Nash A., Aghlara-Fotovat S. (2021). The surface topography of silicone breast implants mediates the foreign body response in mice, rabbits and humans. Nat. Biomed. Eng..

[19] Huemer G.M., Wenny R., Aitzetmüller M.M., Duscher D. (2018). Motiva ergonomix round silksurface silicone breast implants: Outcome analysis of 100 primary breast augmentations over 3 years and technical considerations. Plast. Reconstr. Surg..

[20] Atlan M., Nuti G., Wang H., Decker S., Perry T.A. (2018). Breast implant surface texture impacts host tissue response. J. Mech. Behav. Biomed. Mater..

[21] Glicksman C., Wolfe A., McGuire P. (2025). The Study of the Safety and Effectiveness of Motiva SmoothSilk Silicone Gel-filled Breast Implants in Patients Undergoing Primary and Revisional Breast Augmentation: 5-Year Clinical Data. Aesthet. Surg. J..

[22] Aitzetmüller-Klietz M.L., Yang S., Wiebringhaus P., Wellenbrock S., Öztürk M., Kückelhaus M., Hirsch T., Aitzetmüller-Klietz M.M. (2023). Complication Rates after Breast Surgery with the Motiva Smooth Silk Surface Silicone Gel Implants—A Systematic Review and Meta-Analysis. J. Clin. Med..

[23] Adelson D., Singolda R., Haran O., Madah E., Barsuk D., Barnea Y. (2023). Our Experience Using Round Nano-Surface Ergonomix Implants for Breast Reconstruction: A Single-Center Retrospective Study. Aesthet. Surg. J..

[24] Kaplan H.Y., Rysin R., Zer M., Shachar Y. (2023). A single surgeon’s experience with Motiva Ergonomix round SilkSurface silicone implants in breast reconstruction over a 5-year period. J. Plast. Reconstr. Aesthet. Surg..

[25] Dueck A.C., Mendoza T.R., Mitchell S.A., Reeve B.B., Castro K.M., Rogak L.J., Atkinson T.M., Bennett A.V., Denicoff A.M., O’Mara A.M. (2015). Validity and Reliability of the US National Cancer Institute’s Patient-Reported Outcomes Version of the Common Terminology Criteria for Adverse Events (PRO-CTCAE). JAMA Oncol..

[26] Koslow S., Pharmer L.A., Scott A.M., Stempel M., Morrow M., Pusic A.L., King T.A. (2013). Long-term patient-reported satisfaction after contralateral prophylactic mastectomy and implant reconstruction. Ann. Surg. Oncol..

[27] Casella D., Bernini M., Bencini L., Roselli J., Lacaria M.T., Martellucci J., Banfi R., Calabrese C., Orzalesi L. (2014). TiLoop^®^ Bra mesh used for immediate breast reconstruction: Comparison of retropectoral and subcutaneous implant placement in a prospective single-institution series. Eur. J. Plast. Surg..

[28] Montemurro P., Papas A., Hedén P. (2017). Is Rotation a Concern with Anatomical Breast Implants? A Statistical Analysis of Factors Predisposing to Rotation. Plast. Reconstr. Surg..

[29] Guyomard V., Leinster S., Wilkinson M. (2007). Systematic review of studies of patients’ satisfaction with breast reconstruction after mastectomy. Breast.

[30] Marques M., Brown S.A., Oliveira I., Cordeiro M.N.D.S., Morales-Helguera A., Rodrigues A., Amarante J. (2010). Long-term follow-up of breast capsule contracture rates in cosmetic and reconstructive cases. Plast. Reconstr. Surg..

[31] Momoh A.O., Ahmed R., Kelley B.P., Aliu O., Kidwell K.M., Kozlow J.H., Chung K.C. (2013). A Systematic Review of Complications of Implant-based Breast Reconstruction with Prereconstruction and Postreconstruction Radiotherapy. Ann. Surg. Oncol..

[32] Mangialardi M.L., Salgarello M., Cacciatore P., Baldelli I., Raposio E. (2020). Complication Rate of Prepectoral Implant-based Breast Reconstruction Using Human Acellular Dermal Matrices. Plast. Reconstr. Surg. Glob. Open.

[33] Doren E.L., Pierpont Y.N., Shivers S.C., Berger L.H. (2015). Comparison of allergan, mentor, and sientra contoured cohesive gel breast implants: A single surgeon’s 10-year experience. Plast. Reconstr. Surg..

[34] Serletti J.M., Fosnot J., Nelson J.A., Disa J.J., Bucky L.P. (2011). Breast reconstruction after breast cancer. Plast. Surg. Complet. Clin. Masters PRS Breast Reconstr..

[35] Casella D., Fusario D., Pesce A.L., Marcasciano M., Lo Torto F., Luridiana G., De Luca A., Cuomo R., Ribuffo D. (2023). Portable Negative Pressure Wound Dressing in Oncoplastic Conservative Surgery for Breast Cancer: A Valid Ally. Medicina.

[36] Hong P., Kim S.S., Jeong C., Hwang S.H., Kim T.S., Park J.H., Song Y.K. (2021). Four-Year Interim Results of the Safety of Augmentation Mammaplasty Using the Motiva Ergonomix^TM^ Round SilkSurface: A Multicenter, Retrospective Study. Aesthet. Plast. Surg..

[37] Casella D., Rocco N., Luridiana G., Marcasciano M., Zerini I., Sordi S., Neri A., Catanuto G., Ferrando P.M., Kaciulyte J. (2025). Enhancing Breast Reconstruction with Bovine Pericardium: A Preliminary STEP (Surgical Techniques and Efficacy in Pericardium Use) Towards Improved Outcomes. J. Clin. Med..

