# Supplementary figures and images for: Ergonomic Implants: A Single Centre Experience in Post-Mastectomy Breast Reconstruction
Source: Medicina (Kaunas). 2026 May 31;62(6):1064. doi: 10.3390/medicina62061064 (PMC13302850; doi:10.3390/medicina62061064)

**Supplementary File S1.** Details of patient’s enrolment

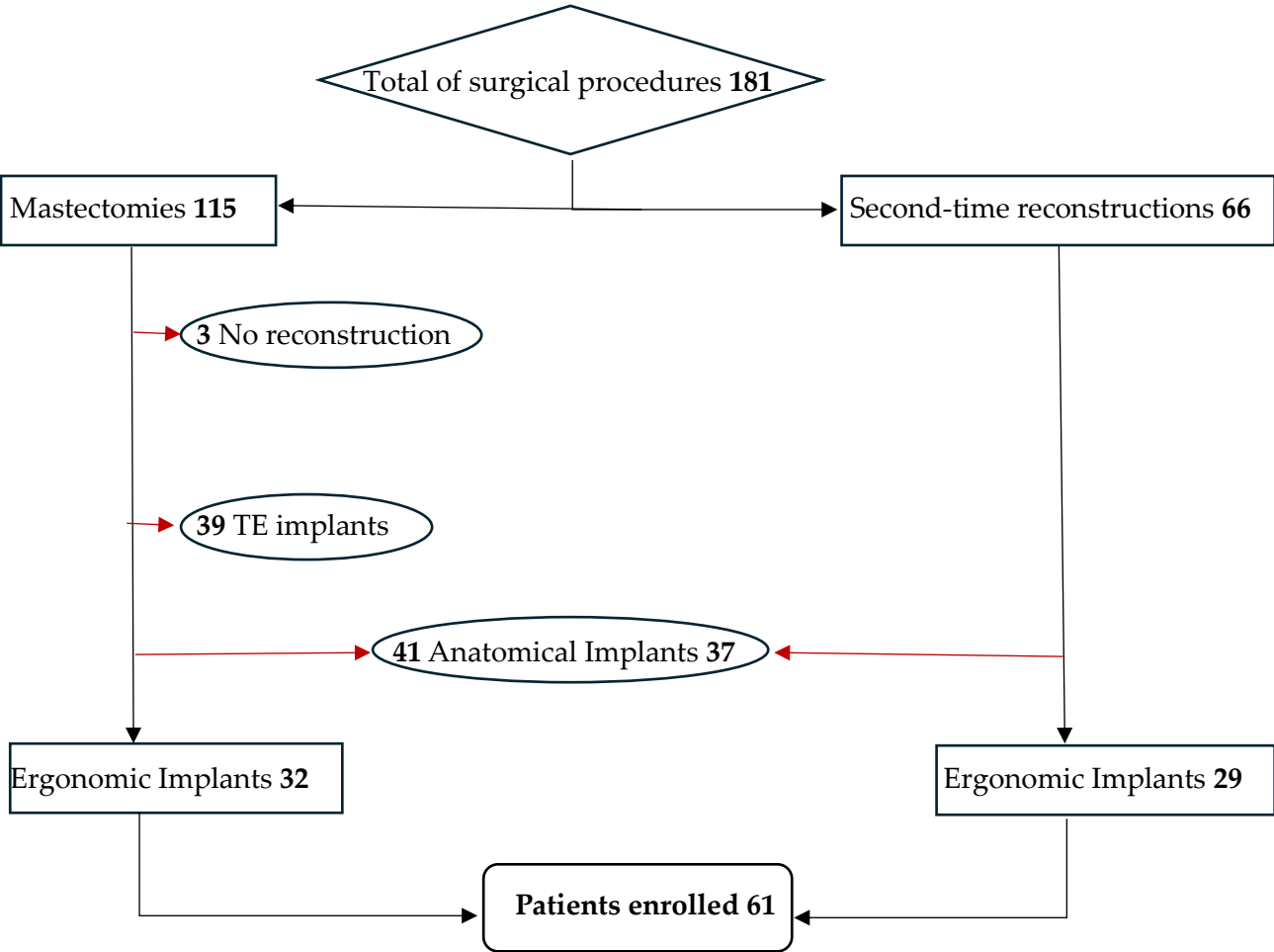

Supplement: Supplementary file 1 [file medicina-62-01064-s001.zip › medicina-4228072-supplementary.pdf]
